# Do media events still unite the host nation’s citizens? The case of the Tokyo 2020 Olympic Games

**DOI:** 10.1371/journal.pone.0278911

**Published:** 2022-12-12

**Authors:** Takeshi Sakaki, Tetsuro Kobayashi, Mitsuo Yoshida, Fujio Toriumi

**Affiliations:** 1 Institute for Future Initiatives, The University of Tokyo, Tokyo, Japan; 2 Department of Media and Communication, City University of Hong Kong, Kowloon Tong, Hong Kong; 3 Faculty of Business Sciences, University of Tsukuba, Tokyo, Japan; 4 School of Engineering, The University of Tokyo, Tokyo, Japan; University of Glasgow, UNITED KINGDOM

## Abstract

The Olympic Games are a typical media event and are seen as a festive occasion that monopolizes people’s attention through the mass media. The Games and their media coverage have a predetermined schedule that enhances the nation’s sense of unity by placing a temporary truce on political conflicts. Governments, especially those of Olympic host countries, tend to take advantage of this effect to garner support for their own policies. However, the effects of such media events may be weakening owing to changes in the media environment and increasing political polarization. Examining the 2020 Tokyo Olympic Games, this case study analyzes a large amount of Twitter data to probe Japanese social media users’ attitudes toward the Olympic Games and the relationship of these attitudinal changes with their attitudes toward the political leadership of the prime minister. The results showed that previously negative attitudes toward the Olympic Games improved as people enjoyed the event. However, this positive shift did not appear to be associated with their attitudes toward the prime minister. Users’ political predispositions strongly determined their attitudes toward the Olympic Games, indicating that the Olympic Games as a media event had limited implications for support for the administration.

## Introduction

The Olympic Games are not only a sporting event but also a typical media event with profound political implications [1–3]. According to Dayan and Katz [2], a media event is a major event that is planned in advance, attracts many people, and is broadcast live by the media (especially on television). Media events are classified into three types: contests, conquests, and coronations, and the Olympic Games are a typical example of the contest type. Because media events are announced and broadcast live in real time, they encourage viewers to interrupt their daily lives and participate in the festive experience. In addition, because the organizers of media events are established authorities and the events are ceremonial in nature, they have an aspect of ritualistic efforts to resolve domestic conflicts and restore order in the host countries. The media event thus requires at least a temporary cessation of hostilities, much like the tradition of the Olympic Truce in ancient Greece.

Through such ritualistic features and ubiquitous viewing and participatory experiences, media events are expected to enhance national identity. If the people become enthusiastic and develop a sense of community through their exposure gained from the mass media coverage of events, the regime can call a temporary truce on domestic political conflicts, deflect dissatisfaction away from the regime, and stabilize the administration [4–6]. This is why the Olympic Games have been used to assert particular national ideologies beyond mere sporting events.

However, such media events are becoming less effective in unifying the public, easing domestic conflicts, or stabilizing regimes through shared, festive mass media experiences. Media events have only been able to suspend domestic conflicts and curb political cynicism through collaboration between the International Olympic Committee (IOC), host governments, broadcasters, and mass audiences [5, 6]. However, political polarization in many parts of the world makes it increasingly difficult to achieve consensus on the importance of specific media events. For example, the Watergate hearings were a typical contest-type media event, but the US at the time was less politically polarized than it is today. In contrast, the more polarized impeachment of President Clinton in the late 1990s did not become a media event because Democrats believed that the impeachment was a political maneuver orchestrated by Republicans [7]. In addition, the rapidly changing media environment has limited the power of media events. Mass media coverage alone of the Olympic Games will be less effective in promoting social integration as it has been directed at audiences fragmented by social media use. Social media users selectively consume content based on their own interests, and it has become difficult for the mass media to monopolize the public’s attention. This means that the scale of media content sharing is reduced, so its effectiveness in fostering national identity and a sense of community also diminishes.

Despite this trend, modern nation-states still use the Olympic Games to promote their regime, even though the power of media events has generally diminished. The 2008 Beijing Olympic Games were aimed at strengthening national identity by demonstrating China’s development, both domestically and internationally [8]. This objective proved successful to a certain extent. For example, Chinese national identity was significantly enhanced in Hong Kong in 2008, which had been returned to China nearly 10 years previously [9]. However, when protests against human rights violations in Tibet arose in conjunction with the torch relay in Paris and London, anti-Western nationalism emerged in online discussions, contrary to the Chinese government’s intention to use the Olympic Games to showcase China’s peaceful rise [10, 11]. Thus, it is becoming increasingly difficult to achieve desired effects that are convenient for the regime, even when the Olympic Games are held in an authoritarian state with strong control over all media channels.

### The context of the Tokyo Olympic Games

The Tokyo Olympic Games in the summer of 2021 took place at a time when this media event was losing its power to ameliorate conflicts within the host country and enhance national identity. In fact, even before the Games were held, various clashes over values and ideologies erupted. For example, the head of the Tokyo organizing committee resigned after he was criticized for making sexist remarks about women [12], and the creative director of the opening ceremony was forced to resign after it was revealed that he had made ridiculous remarks about the appearance of a female celebrity [13]. Moreover, the musician who was supposed to compose the music for the opening ceremony resigned after it was revealed that he had bullied his disabled classmates at school [14]. In addition, a former comedian who was the show director was dismissed because of his past comedic skits making fun of the Holocaust [15]. These incidents show that the divisions in values over feminism, minority rights, and historical awareness were not alleviated by the Olympic Games, but rather became salient, and the media event could not place a temporary truce on domestic conflicts.

Furthermore, as mentioned above, the diversification of the media environment has meant that the general public does not see only mass media coverage. In Japan, young people are increasingly not watching television and turning to video applications such as YouTube and TikTok to watch what they want [16]. Of course, Japanese people knew that the Olympic Games were going to be held, but before and during the Games there was no situation in which a small number of mass media outlets could entirely paint the media space in Olympic colors and force the entire nation to pay attention, regardless of whether they liked sports. Owing to this diversification of values and changes in the media environment, it was difficult for the Japanese government to use the Olympic Games as a media event to increase the sense of unity among the people and to divert criticism away from the administration.

Nevertheless, the Japanese government appeared to pin its hopes on the Olympic Games to lift its popularity. As the Olympic Games were underway, a strong correlation between the spread of the new coronavirus and the approval rating of the cabinet emerged in Japan [17]. That is, the more people became infected with the virus, the lower the approval rating of the Suga cabinet became. Against this backdrop, the government was keen to use the Olympic Games as an opportunity to turn around the government’s approval rating. For example, the former Chief Cabinet Secretary of the ruling Liberal Democratic Party, Takeo Kawamura, said, “The efforts of the Japanese athletes in the Olympics will be a great source of strength for us” and “[i]f there were no Olympics, the people’s dissatisfaction would be more and more directed against our administration, and we would have to fight a tough election.” This remark suggests that the ruling parties were planning to use the Olympic Games to regain the support of the cabinet and fight the post-Olympic elections to their advantage. However, according to polling data, opposition to the Olympic Games has consistently outweighed support for them [18]. Most of the reasons for opposition were concerns and dissatisfaction over the government’s handling of the pandemic. It was suspected that behind the government’s decision to go ahead with the event despite strong opposition, there was a wishful expectation that once the event started, people would eventually enjoy the Games, and that this would revitalize support for the government [19]. In fact, the plan seemed to have worked. The opening ceremony attracted relatively significant attention, and after a series of medals were won by Japanese athletes, the Japanese audience began to enjoy the Games. The viewership rating for the opening ceremony was 56.4%, close to that of the 1964 Tokyo Olympic Games.

These theoretical arguments and the contextual background raise empirical questions: Did enjoyment of the 2021 Tokyo Olympic Games mitigate anxiety and dissatisfaction with the government of Japan? Did those who opposed their holding enjoy the Games when they started, as media event theory predicted and as the Japanese government hoped? Did this alleviate dissatisfaction with the government? Alternatively, as recent critics of media event theory suggest, did the Olympic Games no longer unify the nation and enhance national identity, or did the growing discontent during the pandemic go unrelieved by the Olympic Games? In addition to the perspective of the political use of sporting events by governments, an answer to this empirical question would also make a significant theoretical contribution by testing the contemporary relevance of media event theory.

This study uses Twitter data to analyze the research question of whether enjoying the Olympic Games alleviated anxiety and dissatisfaction with the government. There are several advantages to using data from Twitter rather than public opinion polls to examine this research question. First, while it is possible to track approximate within-individual changes in public opinion polls using a panel design that obtains multiple data points from the same respondents, it is difficult to track the changes in detail because the maximum number of waves is limited to several around the time of the Olympic Games. In this respect, Twitter data have the advantage that it is easy to track changes in a time series. In addition, Twitter data can measure spontaneous expressions of opinions and emotions, rather than the attitudes that are constructed from poll responses to fixed questions. On the other hand, Twitter data are less representative than poll data, and because questions about attitudes toward the administration and the Olympic Games are not asked directly, it is necessary to estimate them.

Despite its lack of representativeness, analyzing Twitter data is warranted because of at least two processes whereby Twitter plays a unique role in shaping public opinion. First, visualized Twitter data has feedback effects on public opinion through the mass media and election campaigns. Journalists report public sentiment based on Twitter data and use it to create horse-race narratives of elections [20, 21]. Similarly, election campaigners use Twitter data as an indicator of public opinion and design the campaign strategy, thereby seeking to influence public opinion and to win elections [22]. Second, public opinion is influenced by the perceived opinion climate on Twitter, even without mediation by journalists or election campaigns. As an illustration, if the distribution of perceived opinion on Twitter leads people to believe they are in the minority, their public expression of opinion may be suppressed by a spiral of silence [23, 24]. In light of the emergence of these new dynamics of public opinion via social media, there are significant implications for analyses of changes on Twitter.

## Results

### Data

We used the Twitter API to collect Japanese-language tweets about the Olympic Games and Prime Minister (hereafter PM) Suga. Because this study used only publicly available data and did not involve human subjects, it was exempt from ethical review by the Institutional Review Board in light of the authors’ institutional guidelines. The focus of our analysis is whether a change in attitude toward the Olympic Games is linked to a change in attitude toward the Suga administration. Therefore, the key variables are attitudes toward the Olympic Games and PM Suga. The collected tweets were divided into the periods before the opening of the Olympic Games (July 1–22, 2021), during the Games (July 23–August 8, 2021), and after the closing (August 9–September 2, 2021). This makes it possible to answer questions such as whether people who were previously opposed to the Olympic Games changed their attitude toward PM Suga while enjoying the Games. The keywords used to collect tweets about the Olympic Games were “オリンピック” (Olympic Games) or “パラリンピック” (Paralympic Games) OR “五輪” (Olympic Games) or “聖火” (Torch) or “オリパラ” (Olympic Games and Paralympic Games). The keywords used to collect tweets about PM Suga were “スガ” (Suga) or “菅” (Suga) or “総理” (Prime Minister) or “首相” (Prime Minister). The tweets used in this study are released in accordance with Twitter’s Developer Agreement and Policy (https://doi.org/10.5281/zenodo.7097638).

The numbers of original tweets and their retweets for each period are shown in Table 1. Because the numbers of retweets are much greater than those of original tweets, the inclusion of the original tweets has little effect on the subsequent analyses (hereafter, “retweets” include the original tweets). Olympic Games-related tweets were consistently more frequent than those about PM Suga, and tweets about the Olympic Games were especially frequent during the Games.

**Table 1:** Number of tweets.

Target	Before Olympics	During Olympics	After Olympics
Olympics	15,974,582 (82.29%)	25,712,551 (86.32%)	11,189,684 (69.82%)
PM Suga	3,438,799 (17.71%)	4,073,872 (13.68%)	4,836,926 (30.18%)
Total	19,413,381 (100%)	29,786,423 (100%)	16,026,610 (100%)

*Note*: Numbers include original tweets and retweets.

### Changes in attitudes toward the Olympic Games

**Fig 1:**
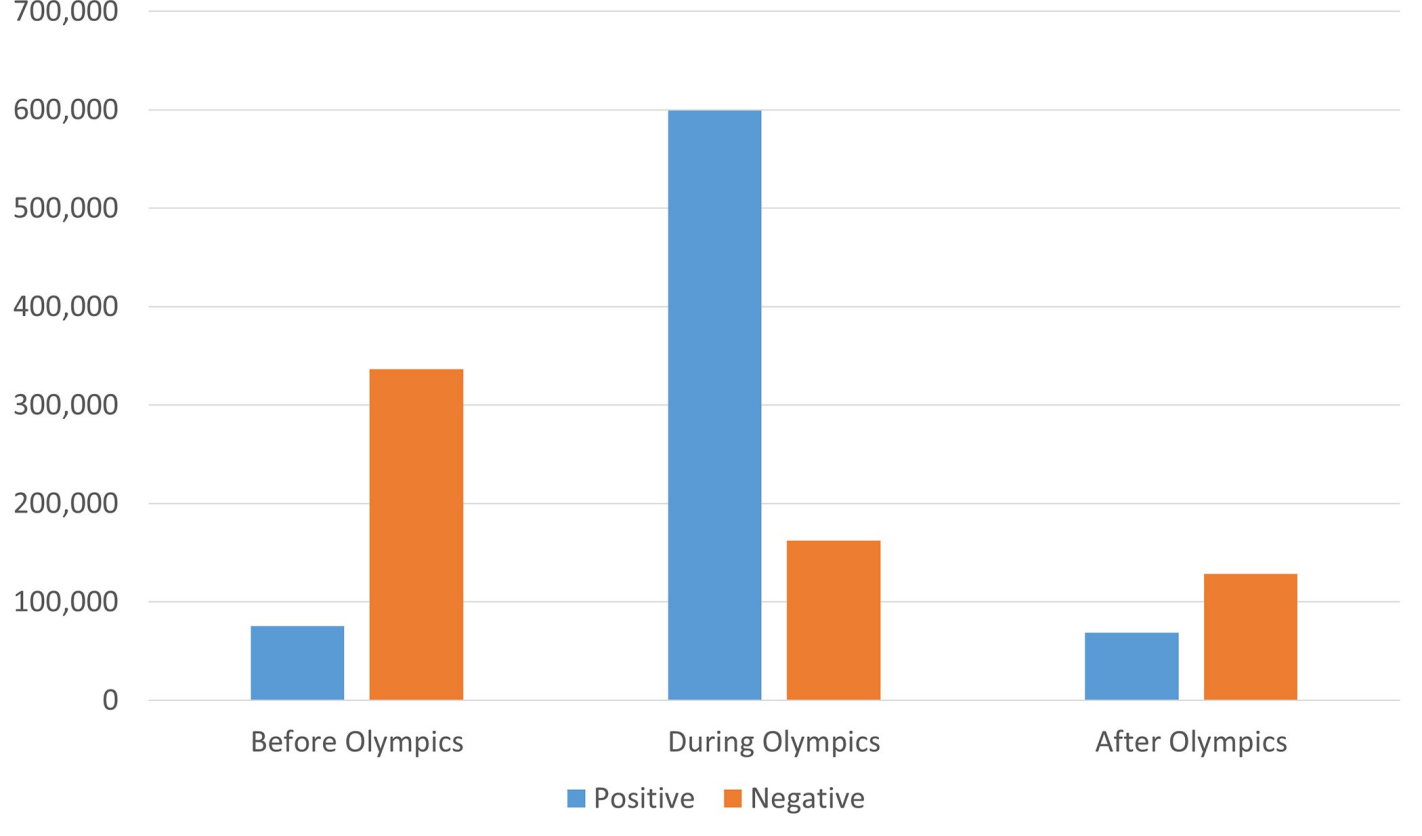
Number of users who posted positive/negative (re)tweets about the Olympic Games in each period.

First, based on the numbers in Table 2, Fig 1 plots the number of users who retweeted positive/negative tweets about the Olympic Games during each period (see the Methods section for an explanation of how Table 2 was created). Before the start of the Games, the number of accounts posting negative retweets accounted for 20.0% in the entire period (336,495/1,680,168), but during the Games this decreased to 4.9% (162,112/3,250,401). On the other hand, the number of accounts posting positive retweets increased from 4.5% (75,321/1,680,168) before the Games to 18.4% (599,275/3,250,401) during the Games. These results indicate that the overall negative reactions before the start of the Games improved significantly after they started.

**Table 2:** Number of users (including duplicates).

		Before Olympics	During Olympics	After Olympics
Olympics	Positive	75,321 (4.48%)	599,275 (18.44%)	68,836 (4.41%)
	Negative	336,495 (20.03%)	162,112 (4.99%)	128,411 (8.23%)
	Total	1,680,168 (100%)	3,250,401 (100%)	1,561,181 (100%)
PM Suga	Positive	45,457 (10.63%)	24,505 (5.38%)	42,259 (7.77%)
	Negative	103,158 (24.13%)	131,065 (28.76%)	111,346 (20.48%)
	Total	427,595 (100%)	455,675 (100%)	543,743 (100%)

*Note*: Because there are tweets that are neither positive nor negative, the sum of “Positive” and “Negative” tweets does not equal the total. Because some users retweeted both positive and negative tweets, the numbers in Table 2 include duplicates.

The strength of our data lies not only in showing these overarching trends but also in the ability to track changes in attitude toward the Games within each user. In the following analysis, “P” refers to accounts that have retweeted positive tweets about the Olympic Games, “N” refers to accounts that have retweeted negative tweets, and “–” refers to accounts that have retweeted tweets that are Games-related but neither positive nor negative. Based on these classifications, we denote accounts that changed cluster affiliation from negative to positive from before to during the Olympic Games as “NP,” accounts that remained negative as “NN,” accounts that changed from positive to negative as “PN,” and accounts that remained positive as “PP.” We also denote accounts that retweeted positive tweets beforehand but did not retweet positive or negative tweets during the Olympic Games as “P–.” Furthermore, to capture attitudinal changes clearly, accounts that posted both positive and negative tweets during the same period were excluded from the analysis. The results of this categorization of attitudinal changes at the user level from before to during the Games are shown in Table 3.

**Table 3:** Changes in attitudes toward the Olympic Games at the individual level.

Type	Number of Users
NN	67,518 (8.01%)
NP	71,944 (8.53%)
PN	414 (0.05%)
PP	28,819 (3.42%)
N–	171,684 (20.36%)
P–	20,739 (2.46%)
–N	38,886 (4.61%)
–P	443,218 (52.56%)
Total	843,222 (100%)

As indicated by the number of accounts labeled “NP,” 71,944 users, or 23% of the 336,495 users (see Table 2) who expressed negative sentiments beforehand came to show positive attitudes during the Games. On the other hand, among the users who showed positive attitudes before the opening ceremony, only 414 showed a negative attitude after the Games opened. Therefore, it is confirmed that even when examined at the individual level, the attitude change was mostly negative to positive. The main purpose of this study is to analyze whether this turnaround in attitudes toward the Olympic Games has led to a change in attitudes toward the administration, and especially its leader, the prime minister.

### Associations with attitudes toward PM Suga

**Fig 2:**
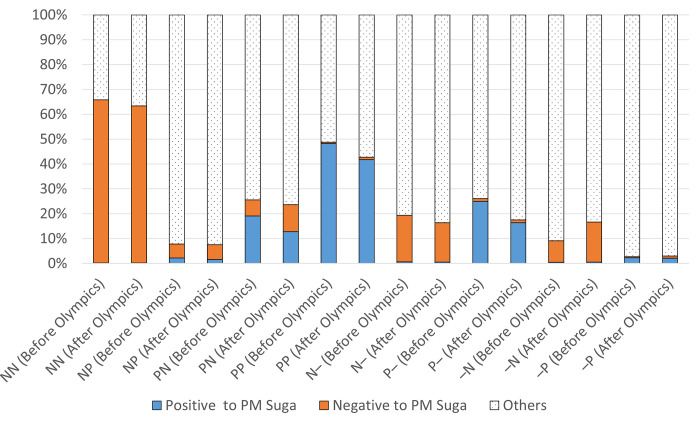
Associations of attitudinal changes toward the Olympic Games and PM Suga.

To examine the associations of attitudes toward the Olympic Games and toward PM Suga, Table 4 and Fig 2 show the percentage of people who retweeted positive/negative messages about PM Suga for each of the eight patterns of users (see Table 3). We use attitudes toward PM Suga before and after the Olympic Games, measured in three stages: before, during, and after the Games (see Table 1). The rationale behind this decision is that if positive attitudes toward the Games are associated with greater approval of PM Suga, then this improvement should gradually emerge during the Games with some time lag and should be most clearly seen in post-Olympic Games attitudes. In other words, this is a pre–post design to see how public opinion changed before and after the intervention (i.e., the Olympic Games).

**Table 4:** Changes in attitudes toward the Olympic Games at the individual level.

	Share of users who retweeted positive tweets about PM Suga		Share of users who retweeted negative tweets about PM Suga	
	Before Olympics	After Olympics	Before Olympics	After Olympics
NN	0.0015	0.0018	0.6564	0.6323
NP	0.0220	0.0159	0.0556	0.0592
PN	0.1908	0.1280	0.0652	0.1087
PP	0.4820	0.4178	0.0060	0.0095
N–	0.0064	0.0051	0.1870	0.1590
P–	0.2497	0.1641	0.0110	0.0113
–N	0.0048	0.0055	0.0864	0.1611
–P	0.0227	0.0212	0.0042	0.0085

Fig 2 shows how the percentage of accounts that retweeted positive or negative messages about PM Suga changed before and after the Olympic Games. For example, “NN (Before Olympics)” shows the distribution of the attitudes of NN users toward PM Suga before the Olympic Games, with about 65% negative (orange bars) and almost none that were positive (blue bars). This distribution did not change after the Games, as indicated by “NN (After Olympics),” indicating that the NN group who were negative toward the Olympic Games before they opened continued to have negative attitudes toward Suga afterward.

Fig 2 shows that positive attitudes toward PM Suga (blue bars) did not increase significantly in any of the patterns of attitude change toward the Olympic Games. The PP group, who had positive attitudes toward the Games before they started includes many users with positive attitudes toward PM Suga before and after the Games. Nevertheless, after the Games, the proportion of users that retweeted positively about PM Suga decreased from 48.20% to 41.78%. More importantly, if support for PM Suga increased because of the Olympic Games, we would expect the increase to be most pronounced for those in the NP group, who were negative beforehand but responded positively during the Games. However, these results showed that they retweeted very little about PM Suga either before or after the Games, and the change in the ratio of positive/negative retweets was negligible. The–P group, which had the highest number of users who expressed no attitudes before the Olympic Games but showed positive attitudes during them, also showed little change in attitude toward PM Suga. The same was true for those in the N–group, which had the next highest number of users, and whose attitudes were negative beforehand and unclear during the Games. They showed little change in attitudes toward PM Suga.

Overall, we found no significant change in attitudes toward PM Suga, regardless of changes in attitude toward the Olympic Games. Therefore, we can conclude that public enjoyment of the Olympic Games did not boost PM Suga’s popularity.

### Political polarization limits the relevance of media events

Why did hosting a typical media event, the Olympic Games, not translate into goodwill toward the prime minister as the administration had hoped? Political polarization in different parts of the world has made it increasingly difficult to reach a consensus on the importance of certain domestic media events. Increasing political polarization promotes selective exposure to politically like-minded information, and as a result, one’s own political attitudes are likely to be reinforced but less likely to change. Thus, if political dispositions entrench one’s view of the prime minister, even if the media event is staged as a national festival and many people enjoy it, this is unlikely to translate into support for the political leader.

**Fig 3:**
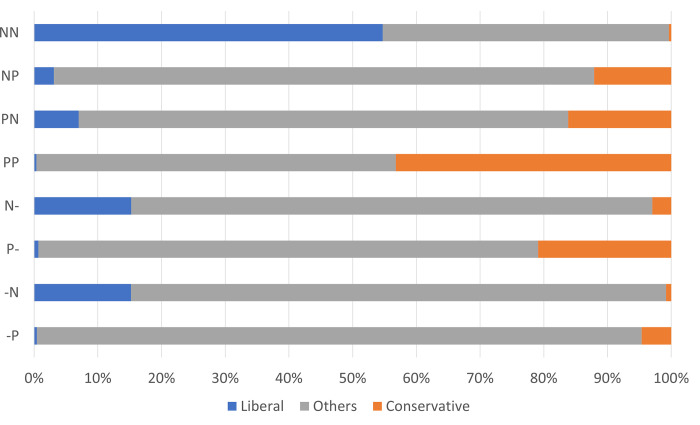
Distribution of political disposition by type of attitude change toward the Olympic Games.

To probe this possibility, we examined how the political disposition of users was related to changes in attitudes toward the Olympic Games. Specifically, we categorized each user’s political disposition into conservative, liberal, and others using the estimation in our previous study [25]. This method estimates the political disposition of each user based on patterns of retweets from February 10, 2019, to October 7, 2020, about former PM Abe, the predecessor of PM Suga. Thus, those who retweeted about the Olympic Games and/or PM Suga were connected to their past political retweets. We assume that the estimated political dispositions had not changed before the Olympic Games because evaluations of PM Abe were polarized between liberals and conservatives [26], making the political dispositions estimated from retweets about PM Abe stable. The distributions of political dispositions for each pattern of change in attitudes toward the Olympic Games are shown in Fig 3.

First, a notable pattern is found in NN and PP: a majority of NN users were estimated to have a liberal political disposition, while more than 40% of PP users were estimated to be conservative. This suggests that those who had consistently negative attitudes toward the Olympic Games before the opening were politically liberal and critical of the government, while those who had consistently positive attitudes were conservative and favorable toward the government. Thus, the changes in attitude (or lack thereof) toward the Olympic Games was closely related to political dispositions.

Second, if we compare the NN and NP groups, the latter has a significantly smaller proportion of liberal users and a slightly larger proportion of conservative users. In addition, the political leanings of the majority of users (i.e., others) in the latter group are not obvious. NP users were critical of the Olympic Games before the opening but enjoyed the media event, and according to media event theory, their attitude toward PM Suga was most likely to improve. We have already seen that this did not happen (Fig 2), but their political disposition is found to be characterized by weak political partisanship. In other words, together with the first point, there is room for attitudinal change toward the Olympic Games only when political disposition is not clear. This trend is also consistent in the comparison between PN and PP users. That is, the PN group has a smaller percentage of conservative users and a slightly larger proportion of liberal users than the PP group. More importantly, the PN group has a larger percentage of users with no clear political disposition (i.e., others). In other words, the attitudes of those with a clear political disposition toward the Olympic Games is decided before the event, and there is little room for change. This is consistent with Dayan’s [27] argument that the effect of media events in mitigating domestic conflicts and fostering national unity is undermined by political polarization.

Third, while there is almost no difference in the distribution of political dispositions when comparing N–and–N retweeters, the percentage of conservative users is larger in the P–group than in the–P group. In other words, attitudes toward the Olympic Games before the event are strongly correlated with political disposition, while the correlation between attitudes toward the Olympic Games after the event and political disposition is weaker.

In general, these analyses show that approval or disapproval of the Olympic Games was decided according to the political disposition in the pre-Olympic stage. Even if people later enjoyed the Games, it was difficult to link this enjoyment to a change in attitude toward the government. Although the Olympic Games are claimed to be politically neutral, the bidding process and hosting of the Olympic Games were planned and executed by the government, which was led by the ruling parties, so naturally there is a strong correlation between political disposition and pre-Olympic attitudes. However, Fig 3 shows that those who have a clear political disposition are less likely to change their prior attitudes even after the Olympic Games begin. In other words, enjoyment of the Games as a media event is strongly determined by political disposition; therefore, changes in attitude toward the Olympic Games occur disproportionately among independents with a weak political disposition. Because those whose attitudes toward the Olympic Games improved as a result of their enjoyment are less likely to be partisans in the first place, it is unlikely that their improved attitudes toward the Olympic Games will translate into higher evaluations of the administration responsible for hosting them (see the S1 File on the relationship between the changes of attitude toward PM Suga and political dispositions).

## Discussion

One of the major consequences of media events such as the Olympic Games has been to create festive excitement among domestic audiences, especially within the host country, and to increase national unity by calling a temporary truce on various conflicts and grievances. This consequence is brought about by the mass media monopoly on broadcasting and the mass media coverage of the Games created by the IOC, host governments, broadcasters, and audiences working together to create a homogeneous social reality [4, 6]. However, with the spread of social media and political polarization, the ability of the mass media to create a uniform social reality has begun to wane, which in turn has begun to limit the power of media events [28]. Nevertheless, as long as individual states host the Olympic Games, the host country’s government still has an incentive to use them to create a favorable public opinion of the regime. Against this background, this study aimed to answer the empirical question of whether hosting the 2020 Tokyo Olympic Games has led to favorable perceptions of the Japanese government and especially its leader, the prime minister.

The short answer to this empirical question is no. When we examined the relationship between positive and negative attitudes toward the Olympic Games and PM Suga using Twitter data, we found no change in attitudes toward PM Suga, regardless of the pattern of attitude change toward the Olympic Games. The 2020 Tokyo Olympic Games had to be postponed for a year owing to the COVID-19 pandemic, and even at the time of the Games in 2021, infections were spreading rapidly in Japan, so there was strong opposition to the Olympic Games. Furthermore, there was widespread dissatisfaction with the government’s attempt to hold the Games despite its inability to control the spread of the virus. In this context, the ruling party attempted to regain support for the government by offering the “circus” of the Olympic Games. In fact, as the government hoped, attitudes toward the Olympic Games improved rapidly after they opened (Fig 1). Nonetheless, there was no evidence that this improvement bolstered support for PM Suga (Fig 2). In addition, when we examined the relationship with political disposition, we found that attitudes toward the Olympic Games were already entrenched in a way that strongly correlates with political disposition even before the opening of the Games. Whether people enjoyed the Olympic Games was also strongly determined by the political disposition. Attitudes toward the Games changed only among independents whose political disposition was unclear, but they did not link this change to attitude toward the prime minister.

It should be noted that the government tried to provide not only the “circus” but also “bread.” Although there was strong opposition to Tokyo’s bid to host the Olympics, the estimated economic ripple effect of approximately 20 trillion yen for Tokyo and approximately 32 trillion yen nationwide, as well as the employment of approximately 1.3 million people in Tokyo and 1.94 million people nationwide, was used to persuade the opposition [29]. Of course, these economic benefits were not expected to be felt immediately during the Games, but the pandemic made the Olympics no-spectators, and the resulting loss of ticket revenues and international spectators’ spending limited the economic effects. Since this study is based on media event theory, it focuses more on the psychological mechanism of the Olympics, but the effect of increased support for the regime due to material benefits was not observed either.

These results suggest that the Olympic Games are losing their power to mask various political divisions by consolidating domestic public opinion in the host country, as recent revisions to media event theory suggest [27, 28]. With the spread of social media, people are becoming more selective in their consumption of content that caters to their interests, rather than sharing content that is provided by a few mass media outlets. Therefore, there is a limit to the efficacy of the mass media in promoting the Olympic Games, no matter how much they try to homogenize the media space [31]. In addition, as seen in the spread of fake news, the ability of the mass media to create a mainstream social reality is waning. These changes in the media environment limit the ability of media events such as the Olympic Games to integrate the domestic public. Furthermore, the political polarization underway in various parts of the world in relation to changes in the media environment is making the boundary between political in-groups and out-groups more salient, making it more difficult for political attitudes to change. The results of this study also showed that attitudes toward the Olympic Games were largely determined by political disposition before the event, and attitudes during the Games were also strongly associated with political disposition. In other words, in a situation where the acceptance or rejection of the festive nature of a media event appears as a political fissure, this is difficult for the media event to conceal. That is, it is quite challenging to use media events to achieve political depolarization.

It is important to note that the attitudes of Twitter users are not representative of public opinion in Japan. Many middle-aged and older people do not use social media and still rely solely on the mass media for information. Among these people, the conventional predictions of media events may be supported. However, it is noteworthy that representative poll data patterns are consistent with those of Twitter found in this study. For example, while only 33% of the respondents favored holding the Olympic Games and 62% called for their postponement or cancellation in a representative poll conducted by the *Asahi Shimbun* newspaper in June 2021, before the Olympic Games [30], the situation was reversed in the poll conducted immediately after they closed, with 56% of the respondents in favor of holding the Olympic Games and 32% opposed [31]. Similarly, in a representative poll conducted by NHK in March and April 2021, before the Games, only 32% of respondents responded that the Olympic Games should be held, while 67% supported postponement or cancellation [32]. However, in a post-Games poll, a total of 78% of respondents reported being “very happy” or “fairly happy” with the Olympic Games, and 75% considered the event to be a success [33]. Furthermore, the fact that PM Suga’s approval rate did not increase during or after the Olympic Games is consistent with the representative poll results. An *Asahi Shimbun* poll conducted in July 2021, just before the Olympic Games, showed 31% support for Suga, while a poll conducted in August, after the Games had ended, showed 28% support [34].

Despite the similarities between the trends we detected in Twitter data and those indicated by representative polls, the unique contribution of this study can only be made by analyzing Twitter data; these data measured attitudes toward the Olympic Games and PM Suga from the same set of Twitter users and traced their changes over time. This analysis would not be possible using polls that measure attitudes toward the Olympic Games and PM Suga at a single point in time. News coverage using Twitter data is increasing in Japan [35, 36]. As the use of Twitter data by news reports and election campaigns becomes more sophisticated, Twitter’s role in revealing the dynamics of public opinion will grow. With that said, we acknowledge that the classification into positive and negative categories is rough, although it captures the most fundamental dimension of human attitudes. More detailed classifications of the discourses related to the Olympic Games and the prime minister are left for future research.

In conclusion, this study applied the framework of media event theory to analyze, using Twitter data, whether the Tokyo 2020 Olympic Games contributed to a positive turnaround in public opinion toward the host country’s government. The results did not support the predictions of conventional media event theory, suggesting that media events no longer substantially unify the public of the Olympic Games host country in the contemporary media environment and political context.

## Methods

### Clustering of tweets

Collected tweets were used to estimate the user’s attitudes toward the Olympic Games and PM Suga. This estimation was performed in two stages. In the first stage, we clustered tweets based on the pattern of retweets. Information on Twitter is disseminated by retweeting, and the users who spread a particular tweet are likely to be interested in it. Therefore, we can infer that those retweeted by similar users have very similar content. For example, a user who opposes the Olympic Games is likely to retweet a tweet that criticizes the Games. Similarly, if a user favors PM Suga, s/he is likely to retweet many tweets reflecting this. Therefore, by using the similarity of users who retweet, we can cluster tweets for each topic (i.e., Olympic Games and PM Suga). In the second step, based on the clusters of tweets obtained, we estimate the attitudes of the retweeters. For example, a user who retweets many tweets from clusters that support PM Suga can be estimated to have a favorable attitude toward him. Specifically, in the first stage, we used the retweet network-based clustering method to cluster 1,000 tweets with the highest number of retweets concerning PM Suga and the Olympic Games in each of the three periods. The effectiveness of this clustering method has been confirmed in previous studies [25, 37–41]. The numbers of detected clusters are shown in Table 5.

**Table 5:** Number of clusters.

	Before Olympics	During Olympics	After Olympics
Olympics	25	21	27
PM Suga	13	11	23

### Merging clusters

This study aimed to gauge the change in users’ attitudes toward the Olympic Games and PM Suga from their patterns of retweets. Therefore, it is necessary to extract clusters of tweets that reflect these views. However, positive attitudes toward the targets are expressed on a variety of topics. As an illustration, tweets applauding the opening ceremony or a Japanese athlete winning a medal are both considered to be positive tweets about the Olympic Games, even though they are semantically different topics. To capture positivity and negativity across these multiple topics, we broadened positive/negative clusters by merging them based on the overlap of those who retweeted the tweets. Specifically, in each period of time, if there was more than a 50% overlap of retweeters in any two positive clusters (e.g., the opening ceremony-positive cluster and the medal-winning positive cluster), the two were merged to form one cluster. In other words, we integrated two positive clusters if many of the retweeters were common to both. By repeating this integration procedure, the clusters were integrated into more general, superordinate positive clusters. The same was done for the negative clusters. The clusters were merged in the order of the total number of retweets, and merging was continued until there were no more clusters to merge. The tweets included in the merged clusters were visually inspected, and the largest clusters with positive and negative content about the Olympic Games and PM Suga were selected, resulting in four clusters (positive and negative clusters about the Olympic Games and PM Suga) in each period.

### Classification of users

Retweeters in the positive/negative clusters for the Olympic Games and PM Suga were defined as accounts with positive/negative attitudes toward the respective targets. The respective numbers of users are shown in Table 2. For example, 427,595 users retweeted tweets about PM Suga before the start of the Olympic Games, of whom 45,457 retweeted positive tweets and 103,058 negative tweets. Because there are tweets that are neither positive nor negative, the sum of “Positive” and “Negative” tweets does not equal the total. Because some users retweeted both positive and negative tweets, the numbers in Table 2 include duplicates.

## Supporting information

S1 File Supporting information for Fig 3. S1 Fig. Distribution of political disposition by type of attitude change toward PM Suga (changes from before to during the Games). S2 Fig. Distribution of political disposition by type of attitude change toward PM Suga (changes from before to after the Games).
(PDF)
