# Supplementary material for: Do media events still unite the host nation’s citizens? The case of the Tokyo 2020 Olympic Games
Source: PLoS One. 2022 Dec 12;17(12):e0278911. doi: 10.1371/journal.pone.0278911 (PMC9744282; doi:10.1371/journal.pone.0278911)
Supplement: S1 File — S1 Fig. Distribution of political disposition by type of attitude change toward PM Suga (changes from before to during the Games). S2 Fig. Distribution of political disposition by type of attitude change toward PM Suga (changes from before to after the Games). (PDF) [file pone.0278911.s001.pdf]

Supporting Information

Figure 3 in the main article, which shows the relationship between changes in attitude toward the Olympic Games and political disposition, indicates that changes in attitude toward Prime Minister Suga were classified based on data from before and during the Olympic Games. The relationship between the classification and political dispositions is illustrated in Figure SI1. The basic trend is the same as in Figure 3. That is, more than 60% of those who were consistently negative toward PM Suga (NN) have a liberal political leaning, while about 70% of those who are consistently positive toward PM Suga (PP) have a conservative political leaning. The results are also consistent with Figure 3 in that the NP and PN people who change their attitudes include a large number of "Others" who have no clear political disposition. Thus, it is suggested that attitudes not only toward the Olympic Games but also toward PM Suga are deeply entrenched in political disposition and are difficult to change. This result implies that the political polarization inherited from PM Abe's time makes it difficult to change attitudes toward PM Suga, which in turn limits the effectiveness of the Games as a media event.

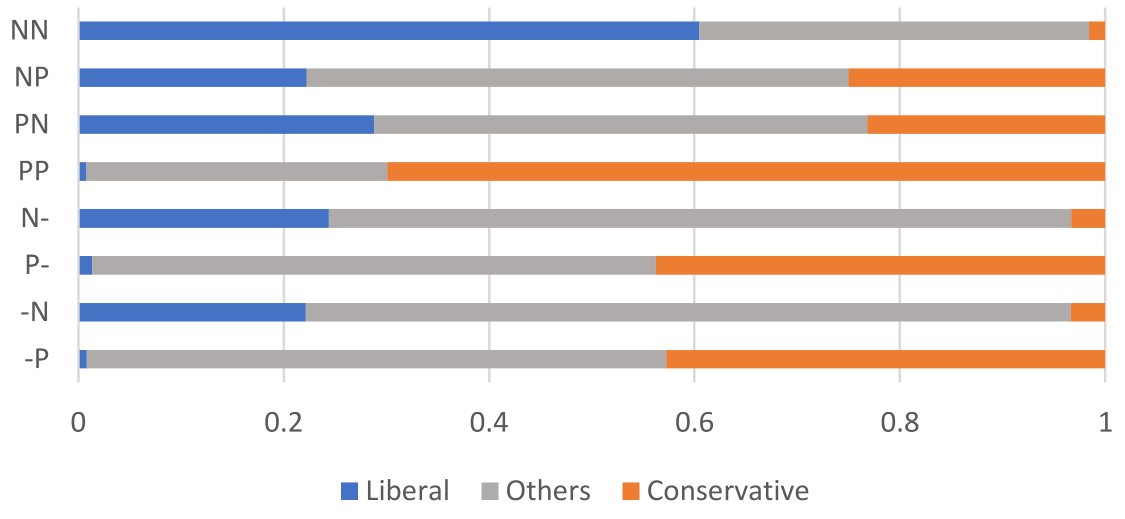

Figure SI1. Distribution of political disposition by type of attitude change toward PM Suga (changes from before to during the Games).

The pattern shown in Figure SI1 does not change significantly when changes in attitudes toward PM Suga are gauged using data gathered before and after the Olympic Games (Figure SI2).

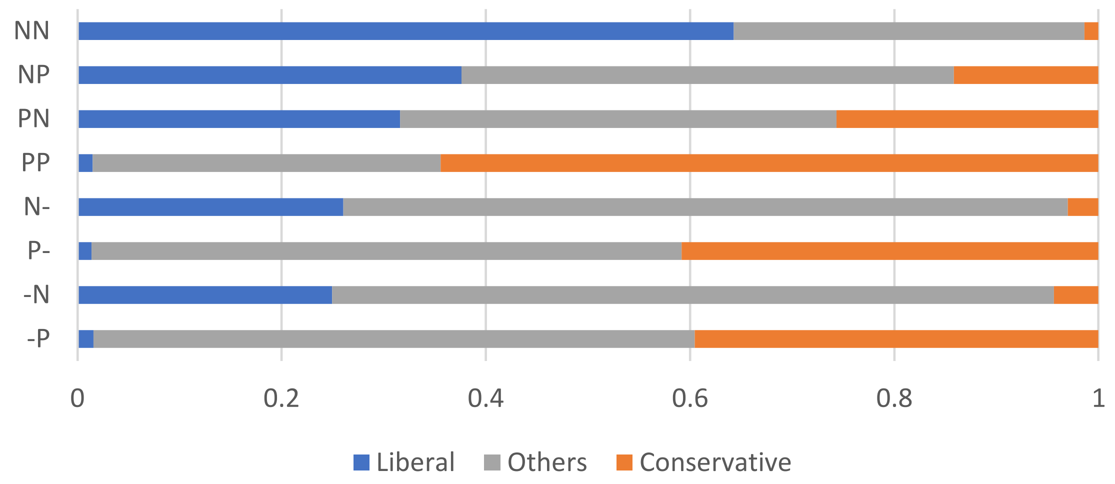

Figure SI2. Distribution of political disposition by type of attitude change toward PM Suga (changes from before to after the Games).
